# Cytotoxicity of Triterpenes from Green Walnut Husks of *Juglans mandshurica* Maxim in HepG-2 Cancer Cells

**DOI:** 10.3390/molecules201019252

**Published:** 2015-10-22

**Authors:** Yuanyuan Zhou, Bingyou Yang, Zhaoxi Liu, Yanqiu Jiang, Yuxin Liu, Lei Fu, Xiaoli Wang, Haixue Kuang

**Affiliations:** 1 College of Pharmacy, Heilongjiang University of Chinese Medicine, Harbin 150040, China; E-Mails: zhouyuanyuan1998@163.com (Y.Z.); ybywater@163.com (B.Y.); liu_zhao_xi@163.com (Z.L.); jiangyanqiu219@163.com (Y.J.); ZYC19901014@126.com (Y.L.); fl0451@outlook.com (L.F.); 2 College of Adult Education, Heilongjiang University of Chinese Medicine, Harbin 150040, China; E-Mail: wangxiaolicando2000@126.com

**Keywords:** *Juglans mandshurica* Maxim, green walnut husks, triterpenoid aglycones, cytotoxic activity, structure-activity relationships

## Abstract

Among the classes of identified natural products, triterpenoids, one of the largest families, have been studied extensively for their diverse structures and variety of biological activities, including antitumor effects. In the present study, a phytochemical study of the green walnut husks of *Juglans mandshurica* Maxim led to the isolation of a new dammarane triterpene, 12β, 20(*R*), 24(*R*)-trihydroxydammar-25-en-3-one (**6**), together with sixteen known compounds, chiefly from chloroform and ethyl acetate extracts. According to their structural characteristics, these compounds were divided into dammarane-type, oleanane- and ursane-type. Dammarane-type triterpenoids were isolated for the first time from the *Juglans* genus. As part of our continuing search for biologically active compounds from this plant, all of these compounds were also evaluated for their cytotoxic activities against the growth of human cancer cells lines HepG-2 by the MTT assay. The results were shown that 20(*S*)-protopanaxadiol, 2α,3β,23-trihydroxyolean-12-en-28-oic acid and 2α,3β,23-trihydroxyurs-12-en-28-oic acid exhibited better cytotoxicity *in vitro* with IC_50_ values of 10.32 ± 1.13, 16.13 ± 3.83, 15.97 ± 2.47 μM, respectively. Preliminary structure-activity relationships for these compounds were discussed.

## 1. Introduction

Globally cancer is the second largest cause of death after cardiovascular disease. More seriously, epidemiological evidence indicates that cancer incidence rates are still increasing in many parts of the world [1]. Drug therapy has an important position in the treatment of malignant tumors, and it is worth noting that discoveries of tumor-resistant pharmacological drugs have mainly resulted from screening of natural products and their analogs such as polysaccharides, naphthoquinones, curcuminoids, and anthocyanins, as well as triterpenoids. Natural antitumor drugs have also proven effective and less toxic for cancer therapy [2]. Recently, some plants from the *Juglans* genus have been studied and recognized for their ability to produce secondary metabolities with anti-tumor properties [3].

**Figure 1:**
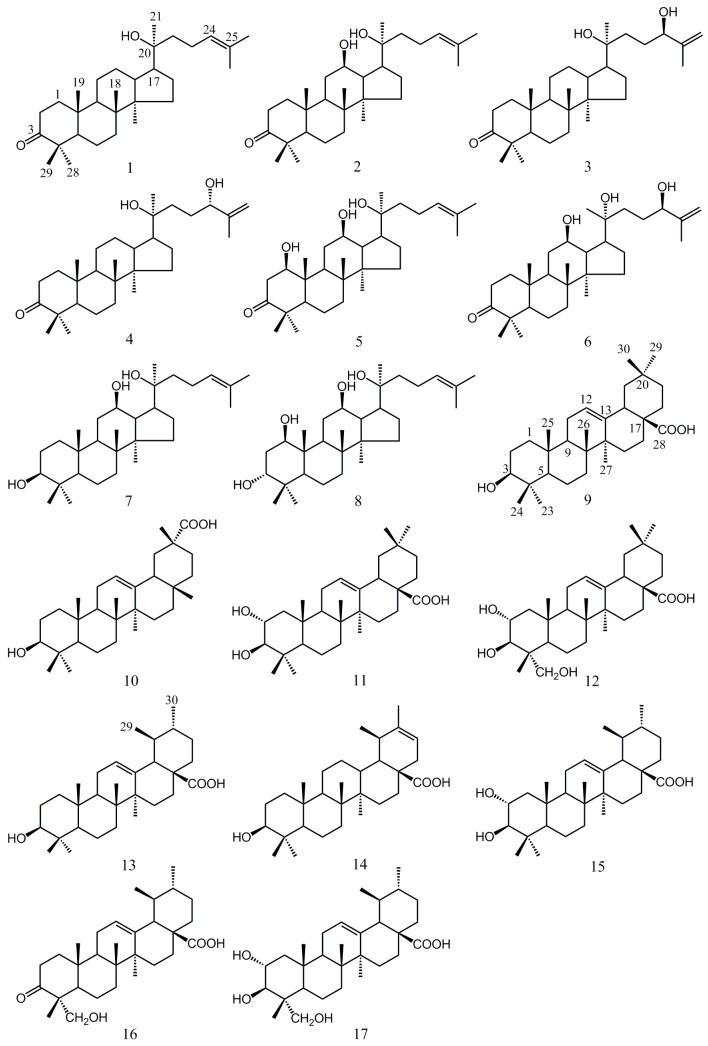
The chemical structures of compounds **1**–**17**.

*Juglans mandshurica* Maxim (GHJ) is the well-known member of the *Juglans* genus which is wildly distributed in the northeast of Asia. Its green husks, shells, leaves and barks all have good antitumor effects [4,5,6]. Considering the better reproducibility of green husk extractions compared with other medicinal parts, we carried out a series of phytochemical and pharmacological studies of green husks of GHJ. Preliminary anti-tumor activity screening study found that the EtOAc and CHCl_3_ fractions from the EtOH extract of GHJ showed significant anti-tumor activities [7] and seventeen compounds including dammarane-type, oleanane- and ursane-type were isolated and identified (Figure 1). These included 20(*S*)-hydroxydammar-24-en-3-one (**1**) [8], 20(*S*)-protopanaxadiol-3-one (**2**) [9], 20(*S*),24(*R*)-dihydroxy-dammaran-25-en-3-one (**3**) [10], 20(*S*),24(*S*)-dihydroxydammaran-25-en-3-one (**4**) [10], 1β,12β,20(*S*)-trihydroxydammar-24-en-3-one (**5**) [11], 12β,20(*R*),24(*R*)-trihydroxydammar-25-en-3-one (**6**), 20(*S*)-protopanaxadiol (**7**) [12], 1β,3α,12β,20(*S*)-tetrol-24-ene-dammar (**8**) [11], oleanolic acid (**9**) [13], 3-epikatonic acid (**10**) [14], 2α-hydroxyoleanolic acid (**11**) [13], 2α,3β,23-trihydroxyolean-12-en-28-oic acid (**12**) [15], ursolic acid (**13**) [16], 3β-hydroxyurs-20-en-28-oic acid (**14**) [17], 2α-hydroxyursolic acid (**15**) [13], 3-oxo-23-hydroxyurs-12-en-28-oic acid (**16**) [18], 2α,3β,23-trihydroxyurs-12-en-28-oic acid (**17**) [19]. Among of them, dammarane-type triterpenoids were isolated for the first time from the *Juglans* genus and a new dammarane triterpene **6** was isolated and its structure elucidated by 1D-, 2D-NMR spectroscopy, HR-ESI-MS and Mosher’s method.

Based on related pharmacological researches and clinical reports, GHJ indeed provides a new alternative treatment strategy against liver cancer. For instance, Ren and Meng reported the use of GHJ drug cocktail therapy in the treatment of liver cancer [20]. Eidi investigated the protective effect of walnut extracts against carbon tetrachloride (CCl_4_)-induced liver damage in rats [21]. Their results indicated that GHJ had an important role in assisting chemotherapeutic drugs to inhibit liver tumor metastasis and acting as a good hepatoprotective and antioxidant agent in attenuating hepatocellular damage. Nowadays, studies on the antitumor effects of GHJ are mostly focused on its naphthoquinones and phenolic compounds, and studies of the activities of its triterpenoids haven’t been reported yet. The aim of this work was to estimate the effect of GHJ triterpenoids on liver cancer and identify potential natural product-based drugs. Thus, the cytotoxic effects of isolated compounds **1**–**7** were evaluated in HepG-2 liver cancer cells using MTT assays. Among these compounds, 20(*S*)-protopanaxadiol (**7**), 2α,3β,23-trihydroxyolean-12-en-28-oic acid (**12**) and 2α,3β,23-trihydroxyurs-12-en-28-oic acid (**17**) exhibited superior cytotoxicity *in vitro*. In addition, preliminary structure-activity relationships for these compounds were discussed.

## 2. Results and Discussion

### 2.1. Isolation and Characterization of Compound **6**

Compound **6** was isolated as colorless crystals from the EtOAc fraction of the ethanol extract of GHJ. The molecular formula was deduced from the HR-ESI-MS (*m*/*z* 475.7352 [M + H]^+^, 497.7304 [M + Na]^+^) and the NMR data to be C_30_H_50_O_4_. The IR spectrum of **6** showed strong hydroxyl absorption bands at 3409 and 3311 cm^−1^, carbonyl group at 1680 cm^−1^ and alicyclic hydrocarbon at 2931 and 2837 cm^−1^.

**Figure 2:**
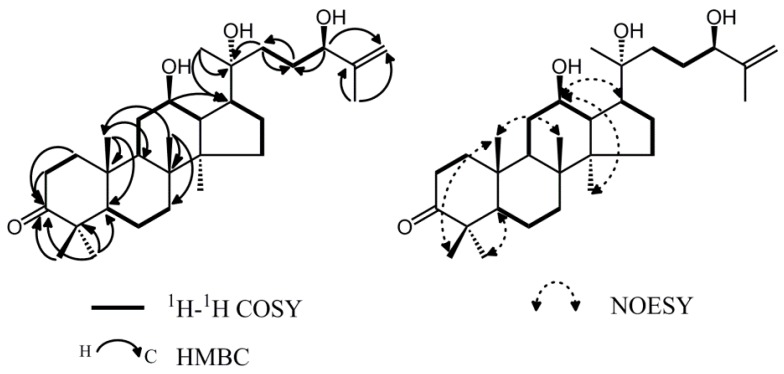
Key ^1^H-^1^H COSY, HMBC and NOESY correlations of **6**.

The ^1^H- and ^13^C-NMR assignments of **6** based on the DEPT and 2D-NMR (COSY, HSQC, HMBC and NOESY) experiments suggested **6** was a dammarane-type triterpenoid (Figure 2, Table 1). The ^1^H-NMR spectrum showed seven methyl signals in the high-field region and a pair of olefinic protons which existed in the form of single peak. The ^13^C-NMR spectrum showed 30 carbon signals. Among them, the characteristic downfield at δ_c_ 217.9 was due to a carbonyl group. The signals at δ_c_ 70.8, 73.6 and 75.3 revealed the presence of three oxygen-bearing carbons. Two olefinic carbons at δ_c_ 147.4 and 110.6 were also assigned. In comparison with ^1^H- and ^13^C-NMR data, it was found that compounds **6** and **3** were similar [10]. The difference was observed at position 12 where the methylene carbon signal at δ_c_ 25.0 in compound **3** disappeared and was replaced with the new methine signal δ_c_ 70.8 in compound **6**. The results suggested that **6** should be the 12-oxo analogue of compound **3**. Moreover, a comparison of the side chain of these two compounds indicated that they have the same planar structure, except that the signals of C-17, C-21, C-22 and C-23 were shifted from δ_c_ 54.3(C-17), 26.4(C-21), 34.6(C-22), 30.1(C-23) to δ_c_ 53.5(C-17), 27.0(C-21), 29.8(C-22), 28.6(C-23), demonstrating the variation of C-20 configuration. Thus, the C-20 configuration of **6** was established to be *R*. Furthermore, Compound **6** showed significant NOE correlations between H-12 (δ_H_ 3.59) and H-17 (δ_H_ 2.05), H-12 (δ_H_ 3.59) and H-30 (δ_H_ 0.86), which indicated that the hydroxyl group at C-12 was oriented on the β-face of the ring system (Figure 2).

**Figure 3:**
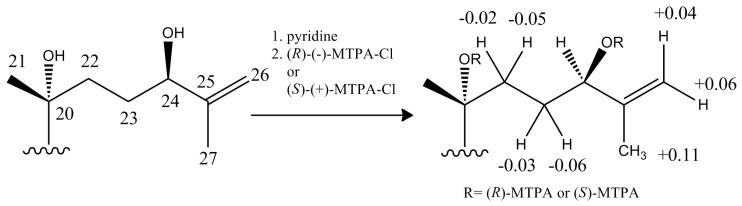
Δδ values (δ*_S_*–δ*_R_*) obtained from (*S*)- and (*R*)-Mosher esters of compound **6**.

The absolute configuration of the chiral carbon atom C-24 was determined by the Mosher method [22]. Treatment of **6** with (*R*)-(−)-α-methoxy-α-(trifluoromethyl) phenylacetyl chloride [(*R*)-(−)-MTPA-Cl] and (*S*)-(+)-α-methoxy-α-(trifluoromethyl) phenylacetyl chloride [(*S*)-(+)-MTPA-Cl] in pyridine yielded a mixture of the 24-(*S*)-Mosher ester and 24-(*R*)-Mosher ester of **6**. These mixtures were separated by semipreparative HPLC to obtain the pure 24-(*S*)-Mosher ester and 24-(*R*)-Mosher ester, respectively. The (Δδ*_S-R_*) chemical shift values are summarized in Figure 3, from which the configuration at C-24 was deduced to be *R*. Thus, the structure of **6** was established as 12β,20(*R*),24(*R*)-trihydroxy-dammar-25-en-3-one.

**Table 1:** 1D- and 2D-NMR data of **6** in CDCl_3_ (δ in ppm, *J* in Hz).

No.	δ_H_ (*J* in Hz)	δ_C_ (from HSQC)	DEPT	HMBC	COSY
1	1.48 (*m*) 1.95 (*ddd*, 15.8, 9.4, 3.0)	39.6	CH_2_	C-2, C-3, C-19	H-2
2	2.45 (*ddd*, 15.8, 4.5, 3.0) 2.53 (*ddd*, 15.8, 9.4, 3.0)	34.1	CH_2_	C-1, C-3, C-10	H-1
3	-	217.9	C		
4	-	47.4	C		
5	1.47 (*m*)	55.2	CH	C-8, C-9, C-19, C-28, C-29	H-6
6	1.54 (*m*)	19.6	CH_2_	C-5, C-9, C-18, C-19	H-5, H-7
7	1.30 (*m*) 1.53 (*m*)	34.1	CH_2_	C-8, C-18	H-6
8	-	39.8	C		
9	1.52 (*m*)	49.4	CH	C-10, C-19	H-11
10	-	36.8	C		
11	1.31 (*m*) 1.82 (*m*)	31.7	CH_2_	C-12	H-9, H-12
12	3.59 (*dt*, 10.4, 5.1)	70.8	CH	C-17	H-11
13	1.75 (*m*)	47.8	CH	C-8, C-12, C-17	H-12
14	-	51.6	C		
15	1.05 (*m*) 1.51 (*m*)	31.0	CH_2_	C-14, C-17	H-16
16	1.26 (*m*) 1.88 (*m*)	26.6	CH_2_	C-17	H-15, H-17
17	2.05 (*m*)	53.5	CH	C-13, C-17, C-20, C-22	H-16
18	0.98 (*s*)	16.0	CH_3_	C-7, C-8, C-10, C-14, C-19	
19	1.01 (*s*)	15.3	CH_3_	C-5, C-8, C-9, C-10	
20	-	73.6	C		
21	1.19 (*s*)	27.0	CH_3_	C-17, C-20, C-22	
22	1.52 (*m*) 1.62 (*m*)	29.8	CH_2_	C-20, C-23	H-23
23	1.62 (*m*) 1.83 (*m*)	28.6	CH_2_	C-22	H-22, H-24
24	4.13 (*m*)	75.3	CH	C-23, C-26	H-23
25	-	147.4	C		
26	4.87 (*s*) 4.98 (*s*)	110.6	CH_2_	C-24, C-27	
27	1.73 (*s*)	18.6	CH_3_	C-24, C-26	
28	1.08 (*s*)	26.7	CH_3_	C-3, C-4, C-5, C-29	
29	1.04 (*s*)	21.0	CH_3_	C-3, C-4, C-5, C-28	
30	0.86 (s)	16.8	CH_3_	C-8, C-14, C-15, C-16, C-18	

### 2.2. Cytotoxic Activity

It was reported that green husks of *Juglans mandshurica* Maxim had an obvious effect on liver cancer. HepG-2 is a kind of human liver cancer cells which are often utilized to assess cytotoxic activity *in vitro*. In this study, we tested the cytotoxicity of compounds **1**–**17**. According to their aglycone moieties these compounds consisted of dammarane type (**1**–**8**), oleanane type (**9**–**12**), and ursane type (**13**–**17**) triterpenes. Our discussion focuses mainly on the relationships between their structures and activities. The results are summarized in Table 2.

**Table 2:** Cytotoxicities of compounds **1**–**17** from *J. mandshurica* Maxim. in HepG-2 cells line.

Comp.	Structural Features	IC_50_ (μM) ^a^	SD ^b^
PC ^c^	metal complex	4.61	0.66
**1**	dammarane-type	NA	-
**2**		NA	-
**3**		62.23	3.06
**4**		91.69	4.92
**5**		NA	-
**6**		58.12	4.22
**7**		10.32	1.13
**8**		76.53	3.39
**9**	oleanane-type	39.42	2.55
**10**		95.5	4.46
**11**		34.80	0.33
**12**		16.13	3.83
**13**	ursane-type	28.69	3.17
**14**		NA	-
**15**		47.22	1.98
**16**		NA	-
**17**		15.97	2.47

^a^ IC_50_, concentration required for inhibiting growth of HepG-2 by 50% (in μM). These results are average results of three experiments; ^b^ SD, standard deviation; ^c^ PC, positive control (cisplatin); NA = not active.

Compounds **1**–**8** share the same basic parent structure with dammarane and their C-17 position side chain exists in an open chain form. Compounds **1**–**6** were similar dammarane-type triterpenoids substituted with a carbonyl group at the C-3 position. However, there were fairly wide variations in cytotoxic activity between them. Compounds **1**, **2** and **5** had no activity with one hydroxyl groups at C-20, double bond at C-24 (25) in the side chain. Compounds **3**, **4** and **6** exhibited relatively effective *in vitro* activity with two hydroxyl groups at C-20, C-24, and a double bond at C-25 (26) in the side chain*.* Based on the comparison between valid and invalid groups, it was discovered that the main structural difference was whether or not to they had a hydroxyl group at the C-24 position of the side chain. Compounds with hydroxyl-substitution had better cytotoxic activity. At the same time, a particularly interesting feature is presented by the elucidation of the configuration of the C-24 asymmetric center. Compounds **3** and **6** of the 24(*R*) series were slightly more effective than **4** of the 24(*S*) series with IC_50_ values of 62.23 ± 3.06, 58.12 ± 4.22 and 91.69 ± 4.92, respectively. The results suggested that the structure and configuration of side chain had an influence on activity. Compounds **7** and **8** both had activity with hydroxyl group at the C-3 position. Compound **8** was weaker than **7** in inhibiting tumor growth, which might be related with the α,β configuration of C-3, as well as the number and position of hydroxyl groups. Further, even though the side chains of compounds **2** and **7** were completely consistent with each other, there showed a significant difference in cytotoxic activity. Compound **2** had no activity and **7** showed the strongest activity (IC_50_ = 10.32 ± 1.13 μM) among of the seventeen compounds. Thus it can be seen that the property and location of the substituents have an important influence on bioactivity. The results indicated that a 3β hydroxyl group was the dominating contributor to cytotoxic activity. Oleanolic acid and its isomer, ursolic acid share similar structural features including a 3β-hydroxyl group, C-28-carboxyl group and Δ^12(13)^-alkene. The results indicated that they had cytotoxic activity against the HepG-2 cells, with ursolic acid (IC_50_ = 28.69 ± 3.17 μM) found more active than oleanolic acid (IC_50_ = 39.42 ± 2.55 μM). Compounds **11**, **12**, **15** and **17**, which all had the presence of the 3β-hydroxyl group, C-28-carboxyl group and Δ^12(13)^-alkene segments similar to the structures of oleanolic acid and ursolic acid, also exhibited good cytotoxic activity. Therefore, it was inferred that a 3β-hydroxyl group, C-28-carboxyl group and Δ^12 (13)^-alkene were the main functional groups responsible for the lower IC_50_ values and better inhibition effects. Among them, it was worth noting that **12** and **17** showed higher inhibitory activities than those of oleanane- and ursane- type triterpenoids on the proliferation of the HepG-2 cells, with IC_50_ values of 16.13 ± 3.83 and 15.97 ± 2.47 μM, respectively. They were entirely consistent with the substituent situation of ring-A (2α,3β,23-trihydroxy substituents). Thus, it was deduced that this structure segments were associated with cytotoxic activity. Compounds **10**, **14** and **16** had a limited or negligible effect on HepG-2 inhibitory activities, which might be attributable to the different substructures in the A-, C- E-rings like introduction of the carbonyl group, migration of the double bond or carboxyl group.

Our evaluation of the abilities of different triterpenoids to inhibit the activity of HepG-2 cells confirmed that the principal structural features for their cytotoxic activity are as above. Despite some dammarane-, oleanane- and ursane- type triterpenoids displayed reasonably high levels of *in vitro* activity, these compounds should also be investigated in further studies whether they could be effective *in vivo*.

## 3. Experimental Section

### 3.1. General Information

^1^H-, ^13^C-NMR, DEPT, HSQC, HMBC and NOESY were recorded on Bruker DPX 400 spectrometer (Bruker, Rheinstetten, Germany). All compounds were dissolved in CDCl_3_ and chemical shifts (δ) are expressed in parts per million (ppm) using TMS as an internal standard. Spin multiplicities are given as s (singlet), d (doublet), t (triplet), dd (double doublet) and m (multiplet). High resolution-electron spray ionization (HR-ESI) mass spectra were run on a Waters LCT Premier XE TOF-MS instrument. Optical rotations were recorded using an Anton Paar-MCP 600 polarimeter (Anton Paar, Graz, Austria). The IR spectra were recorded on a Shimadzu FTIR-8400S spectrometer (Shimadzu, Kyoto, Japan). GC was run on Agilent 7890A Gas Chromatograph System (Agilent Technologies, Santa Clara, CA, USA). Melting points were obtained on with Hoover capillary melting point apparatus (Philadelphia, PA, USA). HPLC chromatograms were obtained on an Agilent Technologies 1260 infinity HPLC system (Agilent Technologies, Waldbronn, Germany) and semi-preparative HPLC (Waters, 515-2414, Milford, MA, USA) was performed using a Hypersil-ODS II column (300 mm × 20 mm i.d., 10 μm, Ylite, Dalian, China). De-ionised water was prepared a Milli-Q system (Milford, MA, USA). HepG-2 cells obtained from Institute of Biochemistry and Cell Biology (Shanghai, China) were grown in Dulbecco’s modified Eagle’s medium (DMEM) (NRH0020, Hyclone, Logan, UT, USA), supplemented with 5% fetal bovine serum and 1% antibiotic mixture comprising penicillin-streptomycin, in a humidified atmosphere at 37 °C with 5% CO_2_. A multiscan microplate reader (Thermo Labsystems, Helsinki, Finland) was used for the MTT assays. The solvents used for open column isolation, such as ethyl acetate, methanol, acetonitrile and chloroform were purchased from Merck (Darmstadt, Germany). Fractions obtained from column chromatography were monitored by thin layer chromatography (TLC) (silica gel 60 F_254_, Merck). MTT and Dulbecco’s modified Dagle’s medium (DMEM) were purchased from Sigma Chemical Co. (St. Louis, MO, USA).

### 3.2. Plant Material

The green husks of *J. mandshurica* were collected in late July 2014 from Changbai Mountains (Jilin, China), and identified by the professor Zhen-Yue Wang. The dried samples were grounded into fine powder (60 mesh), and dried thoroughly in an oven at 40 °C for 3 days.

### 3.3. Extraction and Isolation

The air-dried parts of materials (10.0 kg) were powdered and extracted with 95% EtOH (50 L) at room temperature three times for 3 days each time. The extracts were concentrated and then suspended in H_2_O, followed by successive partitioning with *n*-hexane, CHCl_3_, EtOAc and *n*-BuOH, respectively. The CHCl_3_ extract (183 g) was subjected to silica gel (200–300 mesh) column chromatography (CC), eluted with PE–EtOAc (80:1→1:1, *v*/*v*), to afford eight fractions (Fr1–Fr8). Fraction 4 (22.60 g) was subjected to silica gel (200–300 mesh) CC, eluted with PE–EtOAc (50:1→1:1, *v*/*v*), to give fractions 4a–4e. Compounds **1** (23.2 mg), **3** (4.4 mg) and **4** (15.6 mg) were isolated from fraction 4b and compound **2** (12.1 mg) from fraction 4c by repeated column chromatography over silica gel, eluted with PE–EtOAc (50:1→10:1, *v*/*v*). Fraction 5 (6.60 g) was subjected to silica gel (200–300 mesh) CC, eluted with PE–EtOAc (30:1→8:1, *v*/*v*) to afford fractions 5a–5c. Compounds **8** (22.5 mg), **10** (18.5 mg) were isolated from fraction 5b, eluted with PE–EtOAc (20:1→5:1, *v*/*v*). Fraction 8 (4.13 g) was subjected to silica gel (200–300 mesh) CC, eluted with PE–EtOAc (5:1→1:1, *v*/*v*) to obtain **12** (5.2 mg).

The EtOAc extract (154g) was subjected to silica gel CC eluted with mixtures of PE–EtOAc (15:1→1:1, *v*/*v*) and CHCl_3_–MeOH (30:1→1:1, *v*/*v*) to yield seven fractions (Fr1–Fr7). Fraction 1 (26.20 g) was fractionated with PE–EtOAc (15:1→1:1) afford some subfractions 1a–1c. Compounds **5** (7.3 mg), **6** (16.4 mg), **9** (5.1 mg) and **10** (8.3 mg) were isolated from fraction 1b. Fraction 3(8.54 g) was subjected to silica gel (200–300 mesh) CC, eluted with CHCl_3_–MeOH (20:1→1:1, *v*/*v*) to obtain **7** (6.4 mg), **9** (4.9 mg), **13** (5.2 mg). Fraction 4 (10.10 g) was subjected to silica gel (200–300 mesh) CC, eluted with CHCl_3_–MeOH (10:1→2:1, *v*/*v*) to afford fractions 4a–4d. Subfraction 4c (1.1 g) was subjected to semi-preparative HPLC chromatography (MeOH/H_2_O 40:55, *v*/*v*, flow rate 3 mL/min) to yield compounds **11** (3.3 mg, t*_R_* = 26 min), **15** (3.5 mg, t*_R_* = 33 min) and then subjected to semi-preparative HPLC chromatography (MeOH/H_2_O 50:50, *v*/*v*, flow rate 3 mL/min) to yield compound **14** (4.2 mg, t*_R_* = 30 min). Compounds **16** (8.2 mg), **17** (5.6 mg) were isolated from fraction 4d, eluted with CHCl_3_-MeOH (5:1, *v*/*v*).

### 3.4. Spectral Data

*12β,20(R),24(R)-Trihydroxydammar-25-en-3-one* (**6**). Colorless crystals, mp 210–212 °C; ${\left[\alpha \right]}_{D}^{25}$ +78.5 (*c* 0.25, MeOH); IR (KBr) ν_max_ 3409, 3311, 2931, 2837, 1680 cm^−1^; ^1^H-NMR and ^13^C-NMR data see Table 1; HR-ESI-MS (positive): *m*/*z* 475.7352 [M + H]^+^ (calcd for C_30_H_5__1_O_4_, 475.7331), *m*/*z* 497.7304 [M + Na]^+^ (calcd for C_30_H_50_O_4_Na, 497.7265).

### 3.5. Cytotoxicity Assays

#### 3.5.1. Cell Culture

HepG-2 cell line was maintained in DMEM supplemented with 10% fetal bovine serum (FBS), 100 units/mL penicillin and 100 μg/mL streptomycin (Gibco-BRL, Waltham, MA, USA). The cells were incubated at 37 °C in a humidified atmosphere containing 5% CO_2_ for growth.

#### 3.5.2. Measurement of Cell Proliferation by MTT Assay

Cytotoxicity was measured using the MTT assay [23]. HepG-2 cells were counted in a Neubauer hemocytometer and seeded at 5 × 10^4^/well with a final volume of 100 μL and kept overnight for attachment. To determine the 50% inhibitory concentration (IC_50_) against HepG-2 cells, seventeen compounds and positive control, dissolved in dimethyl sulfoxide (DMSO) and diluted with Dulbecco’s Modified Eagle Medium (DMEM) from 300 to 0.5 μM, were added in 96-well microplates and incubated with cells for 24 h. The optical density (OD) was measured at 570 nm using a multiscan microplate reader. All experiments were performed in triplicate. HepG-2 cells incubated without compounds or DMSO were used as control (100% viability) and wells without cells as blank. Cisplatin was used as a standard drug.

## 4. Conclusions

A new dammarane triterpene was obtained from the green husks of *Juglans Mandshurica* Maxim and identified. Besides, all dammarane-type triterpenoids were described for the first time from the *Juglans* genus. With the aim of exploring natural antitumor sources, three series of seventeen triterpenoids including dammarane-type, oleanane- and ursane-type were tested the cytotoxicity against HepG-2 by the MTT method. At the same time, the structure-activity relation investigation indicated that some structure features like configurations of hydroxyl group, introduction of the carbonyl group, migration of the double bond or carboxyl group had a great effect on the antitumor activity. According to the experimental results, these three compounds **7**, **12** and **17** merit further *in vivo* study potentially leading to the development of anti-liver cancer drugs.
